# Comparing the effectiveness of the unified protocol in combination with an additional mindfulness treatment to the unified protocol alone as treatment for adolescents diagnosed with emotional disorders

**DOI:** 10.47626/2237-6089-2020-0046

**Published:** 2021-02-26

**Authors:** Mahboobeh Maleki, Samad Khorramnia, Aliakbar Foroughi, Shahram Amiri, Sasan Amiri

**Affiliations:** 1 Department of PsychologyFaculty of Psychology and EducationAllameh Tabataba’i UniversityTehranIran Department of Psychology , Faculty of Psychology and Education , Allameh Tabataba’i University , Tehran , Iran .; 2 Department of Clinical PsychologyKermanshah University of Medical SciencesKermanshahIran Department of Clinical Psychology , Kermanshah University of Medical Sciences , Kermanshah , Iran .

**Keywords:** Unified protocol, emotional disorders, additional treatment, mindfulness

## Abstract

**Objective:**

Many adolescents suffer from depressive and anxiety disorders simultaneously and current treatment methods do not put enough emphasis on comorbidity of these disorders. The unified protocol for treating emotional disorders in adolescents is a transdiagnostic therapy which targets mutual fundamental factors. Therefore, the current study aims to compare the effectiveness of the unified protocol alone with the unified protocol combined with mindfulness as an additional treatment in adolescents suffering from emotional disorders.

**Method:**

A quasi-experimental study was conducted with adolescents. The participants had been diagnosed with emotional disorders and were divided into a control group (15 participants) and an experimental group (16 participants). Both groups were offered 14 sessions of therapy. They were assessed at pre-test, post-test, and two-month follow-up. Scales used in the study included the Child Behavior Checklist (CBCL), the Children’s Depression Inventory (CDI), and the Youth Anxiety Measure for DSM-5 (YAM-5).

**Results:**

The results showed that both of the treatment methods effectively reduced adolescents’ emotional problems, but improvements were more significant in the group administered the additional mindfulness program. Among the variables assessed, non-phobic anxiety disorders and depression improved more than specific phobia and behavioral problems. Between-subjects (Group) partial etas for non-phobic anxiety, depression, specific phobia, and behavioral problems were 0.67, 0.50, 0.23, and 0.16, respectively.

**Conclusion:**

According to the findings of this study, additional treatment methods such as mindfulness could increase the effectiveness of the unified transdiagnostic protocol for adolescents (UP-A). The therapeutic implications are discussed.

## Introduction

According to the 5th edition of the Diagnostic and Statistical Manual of Mental Disorders (DSM-5), anxiety and depressive disorders encompass a large range of emotional disorders and are among the most prevalent types of psychiatric illnesses during childhood and adolescence. ^1 , 2^ In Iran, anxiety disorders are the most prevalent disorders among children and adolescents. ^3 , 4^ Studies show there is a high rate of comorbidity between anxiety and depressive disorders; such that 16 to 62 percent of children and adolescents are simultaneously diagnosed with criteria for anxiety and depressive disorders. ^5^ If these problems are left untreated at these ages, they might lead to emotional disorders during adulthood and could even become lifelong. ^6^ Experiencing these symptoms, and also behavioral problems or issues of emotion regulation, could significantly affect the quality of adolescents’ functional performance in education and social communication. ^7^

Evidence-based studies demonstrate the effectiveness and advantages of cognitive-behavioral therapies for treating depression and anxiety symptoms among adolescents, ^8^ but cognitive behavioral therapies (CBTs) have faced serious financial and clinical problems due to high comorbidity of emotional disorders and these illnesses’ mutual symptoms. ^9^ Studying the follow-up results of investigations show that about half of adolescents treated with CBT, experience symptom recurrence. Furthermore, anxious adolescents who suffer from comorbid depression have a slower response to anxiety-specific CBT. ^10^ Actually, evidence suggests that most of these therapeutic approaches focus exclusively on symptoms and on reducing them and do not target the mutual fundamental factors (transdiagnostic factors) that play a role in formation and continuity of a wide range of emotional disorders. ^11^

Therefore, there is an emphasis on utilizing treatment methods which not only concentrate on how different disorders are, but also simultaneously focus on multiple disorders and the factors they have in common; methods which are easy to teach, and financially reasonable to apply. ^12^ With an emphasis on regulation of emotion, the unified protocol for transdiagnostic treatment in adolescents (UP-A) utilizes similar CBTs for a spectrum of emotional experiences and their mutual fundamental factors. The therapeutic methods used in this protocol allow clinicians to make diagnoses and recognize sub-threshold symptoms in the unified program. ^1^

Another treatment used for children’s emotional disorders during recent years is mindfulness. Mindfulness is known as a non-judgmental present-moment awareness which validates any thought, sensation or feeling that enters awareness and accepts it the way it really is. ^13^ Mindfulness-based interventions have been mostly examined in adults, but recently they have been increasingly used and studied at younger ages too. ^14 , 15^ Therefore, considering the problems caused by emotional disorders in adolescents and in response to a lack of sufficient investigations of the effectiveness of the unified protocol in this population, the aim of the current study is to compare the effectiveness of the unified protocol combined with additional mindfulness treatment and the unified protocol applied independently.

## Materials and method

### Participants

**Figure 1:**
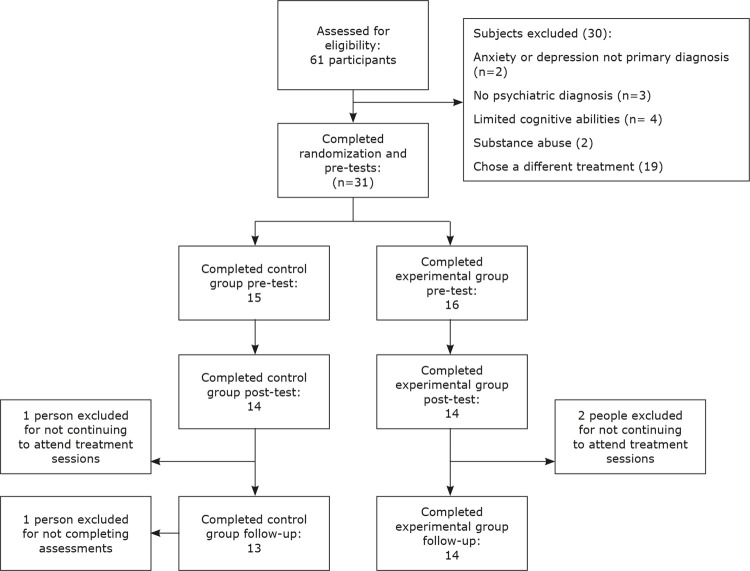
Diagram illustrating numbers of participants in pre-test, post-test and follow-up phases.

The participants were allocated to an experimental group (16 participants) or a control group (15 participants) after screening ( Figure 1 ). Sample size was obtained either from previous studies or calculated using the following formula. ^7^

n=Z1−a/2+Z1−β2s12+s22μ1−μ22n=(10.49)0.862+1.532(5.7−4.1)2=12.88

Considering probable losses from samples, this number was increased to 31. After beginning the treatment, two participants from the experimental group and one from the control group refused to adequately take part in treatment sessions and therefore could not complete the post-test and follow-up assessments. Moreover, one of the control group members was unable to complete the follow-up assessments because of emigration. The average family income was $120 per month.

Three patients from the experimental group and two from the control group were on medication, but their dosages were kept fixed throughout the sessions. At the beginning of treatment, participants were assessed using the Diagnostic Interview for Children and Adolescents (DICA).

### Procedure

This study is a quasi-experimental clinical trial with a control group and an experimental group and was conducted with permission from Kermanshah University of Medical Sciences (KUMS) and after ethics approval code IR.KUMS.REC.1397.178 had been granted. Participants were informed of the trial by announcements at schools and mental health centers in Kermanshah and then referred to Farabi hospital with their parents or caregivers for recruitment to the program. Before beginning the program, caregivers and children both signed informed consent forms. The only therapy offered to the control group was Barlow’s Unified Protocol for Emotional Disorders in Adolescents, whereas members of the experimental group attended the same program and were also assigned to read a self-help book called “Sitting Still Like a Frog: Mindfulness Exercises for Kids (and Their Parents)”. The participants were assessed at pre-test, post-test, and two-month follow-up. Measurements were conducted one day before beginning the sessions, one day after finishing the sessions, and two months after the end of the program. Before starting the intervention, participants were assessed using the Diagnostic interview for children and adolescents (DICA). Teenagers were only included in the study if they were diagnosed with anxiety disorders (generalized anxiety disorder, separation anxiety disorder, social phobia, selective mutism, specific phobia, or anxiety disorder not otherwise specified), depression, or disorders in which anxiety was a main component (obsessive compulsive disorder, posttraumatic stress disorder). Patients were diagnosed with comorbid emotional disorders in addition to their main disorder. Since this research was solely investigating teenagers, only those whose parents were not suffering from clinical disorders were included in the study; those whose parents also had disorders were referred to other treatment programs. Exclusion criteria were: 1: Parent or child unable to read or comprehend the Farsi language while participating in the sessions or completing the questionnaires. 2: Being diagnosed with schizophrenia, pervasive developmental disorders, bipolar disorder type I or II, or any other disorder which prevented the child from understanding the session content or the questionnaire. 3: Suicidal or homicidal ideation. 4: Teenagers whose parents had a history of severe mental illnesses such as psychosis.

Teenagers who did not meet the criteria for the anxiety disorders mentioned above could not be included in the study. Interviews were conducted by an MA psychology student under a psychologist’s supervision. Measurements were conducted by an MA psychology student who was not aware of the aim of the study. The intervention was administered by a psychology student who was trained for the program in workshops run by a faculty member who had translated several third wave psychotherapy books. Additionally, before each session, the session content and the skills needed were reviewed by the supervisor to ensure that the psychology students conducting the sessions met the requirements. During the intervention, students were observed by the supervisor from behind a one-way mirror.

### Intervention

The transdiagnostic treatment administered was based on the Ehrenreich protocol for adolescents. ^1^ This book contains nine chapters covering the following topics: motivating and keeping motivation; identifying emotions and behaviors; introducing emotion-focused behavioral experiments; awareness of bodily sensations; flexibility in thinking; awareness of emotional experiences; exposure to situational emotions; reviewing achievements and planning for the future; and improving parenting style for an emotional adolescent ( Table 1 ). The treatment was delivered as group therapy over 14 sessions. The additional mindfulness treatment which was chosen for this experiment included Eline Snel’s “Sitting like a frog” self-help book. This book consists of mindfulness exercises which are tailored for teenagers and children. The book was given to the experimental group members but not to the control group. To promote commitment to the exercises among the experimental group participants, some of the exercises from the self-help book were done during the session, so that the participants could be engaged in them actively.

**Table 1:** Summary of transdiagnostic treatment protocol

Module	Topic	Content
1	Motivating and keeping motivation	Building a friendly environment, conversation about the key issues and goal setting, motivating the teenager to change
2	Identifying emotions and behaviors	Teaching about emotions and their target, introducing the three parts of each emotion, introducing the cycle of avoidance and other emotional behaviors
3	Introducing emotion-focused behavioral experiments	Introducing the opposite action technique and emotion-focused behavioral experiments, recording emotion and activity levels, emotion-focused behavioral experiments
4	Awareness of bodily sensations	The relationship between intense emotions and bodily sensations, increasing the awareness about bodily sensations, exercises about sensory exposure
5	Flexibility in thinking	Increasing flexible thinking ability, introducing the common cognitive distortions, relating thoughts and behaviors accompanied by problem solving and detective thinking
6	Awareness of emotional experiences	Introducing and practicing present moment awareness, introducing and practicing non-judgmental awareness, general emotional exposure
7	Exposure to situational emotions	Reviewing the skills learnt previously, discussing the rationale of exposure to situational emotions, exposure to situational emotions
8	Keeping achievements	Reviewing the skills and moving forward to future goals, designing a recurrence prevention program
Parents	Improving parenting style for an emotional adolescent	Informing parents of the proper ways of reacting to the teenagers’ distress, introducing four common parenting techniques about emotions and their opposites

### Scales

#### The Youth Anxiety Measure for DSM-5 (YAM-5)

The Youth Anxiety Measure for DSM-5 is a self-report questionnaire for anxiety disorders designed by Muris et al. for detecting anxiety symptoms in children and adolescents. ^16^ It consists of two sections. Part one (28 items) measures the main anxiety disorders in DSM-5 including separation anxiety disorder (6 items), selective mutism (4 items), social anxiety disorder (6 items), panic disorder (6 items), and generalized anxiety disorder (6 items), whereas part two (22 items) consists of five subscales, designed for diagnosis of different types of phobia. All of the items scored on a four-point Likert scale from *never* to *always* . In a study by Simon et al., test-retest reliability for the first section ranged from 0.54 to 0.86 and from 0.73 to 0.89 for the second section. ^17^ According to a study in Iran, internal reliability was from 0.71 to 0.90 for the first section and from 0.65 to 0.91 for the second section. ^18^

#### The Children’s Depression Inventory (CDI)

This questionnaire is a 27-item self-report scale for children aged from 7 to 17 years. It was developed by Kovacs. ^19^ The scale measures the range of depression symptoms such as low mood, being able to enjoy, vegetative functions, self-assessments, and interpersonal behaviors. Each item of the CDI can be scored from 0 to 2 and the total score is between 0 and 54. The validity and reliability of this scale have been reported in studies. ^20 , 21^

#### The Child Behavior Checklist (CBCL)

This checklist consists of 120 questions which assess children aged from 4 to 18 years in six different aspects. The checklist is completed by the child’s parent or another caregiver on the basis of the child’s situation over the last 6 months. Questions are scored from 0 to 2. ^22^ This scale has been normalized in Iran and its internal consistency coefficient was reported to be 0.86 for internalizing and 0.88 for externalizing, with a total coefficient for the whole scale of 0.83. ^23^

#### Diagnostic Interview for Children and Adolescents (DICA)

This scale can be used in structured or semi-structured form. The DICA is suitable for children aged from 6 to 17 years old and takes about an hour or two to complete. The scale covers diagnostic categories such as externalizing behavior disorders, anxiety disorders, depressive disorders, substance abuse disorders, et cetera. ^24^

## Results

The total number of participants in this study was 31. Their demographic characteristics are shown in Table 2 . According to the analysis, there were no significant differences between the two groups of patients in terms of demographic characteristics.

**Table 2:** Demographic characteristics of the subjects

Parameters	Experimental group	Control group
Age (years), mean ± SD	13.06 ± 0.96	13.50 ± 1.09
Gender		
Male	9 (56.25)	10 (62.66)
Female	7 (43.75)	5 (33.33)
Education		
First grade of secondary school	6 (37.5)	5 (33.33)
Second grade of secondary school	4 (25)	3 (20.00)
Third grade of secondary school	6 (37.5)	7 (46.66)

Data presented as n (%), unless otherwise specified. SD = standard deviation.

The patients were diagnosed with emotional disorders and their frequencies of diagnoses at pre-treatment baseline are presented in Table 3 .

**Table 3:** Frequency of Diagnoses at Pretreatment

Diagnosis	Principal diagnosis	Comorbid diagnoses
Generalized anxiety disorder	6 (19.35)	4 (13.33)
Social phobia	5 (16.12)	3 (10)
Major depressive disorder	3 (9.67)	4 (13.33)
Obsessive-compulsive disorder	2 (6.45)	2 (6.66)
Anxiety disorder not otherwise specified	5 (16.12)	7 (14.81)
Panic disorder	2 (6.45)	1 (3.33)
Specific phobia	3 (9.67)	5 (16.66)
Dysthymic disorder	2 (6.45)	2 (6.66)
Post-traumatic stress disorder	3 (9.67)	
Attention-deficit/hyperactivity disorder		2 (6.66)

Data presented as n (%).

Before applying repeated measures ANOVA, some preliminary assumptions were verified using Box’s M test, Mauchly’s test of sphericity, and Levene’s test. Since Box’s M test was not significant for any of the variables, it can be concluded that variance-covariance matrices were homogeneous. Furthermore, the non-significance of any of the variables in Levene’s test indicates equality of between-groups variance and shows that the error variance of the dependent variables is equal in all the groups. Table 4 shows the means and standard deviations of dependent variables in the pre-test, post-test, and follow-up stages.

**Table 4:** Mean and standard deviation of research variables

Variable/Group	Pre-test	Post-test	Follow-up
Depression (CDI)			
Experimental group	66.0) 14.7)	57.0) 21.6)	49.0) 60.9)
Control group	48.0) 30.8)	50.0) 64.7)	51.0) 51.9)
The Child Behavior Checklist (CBCL)			
Experimental group	74.0) 64.35)	74.0) 78.34)	14.1) 92.44)
Control group	04.1) 38.37)	83.0) 85.36)	48.1) 71.43)
Anxiety-I (YAM-I)			
Experimental group	86.0) 14.20)	53.0) 85.18)	94.0) 50.24)
Control group	51.0) 46.21)	73.0) 84.20)	16.1) 14.25)
Anxiety-II (YAM-II)			
Experimental group	49.0) 35.10)	42.0) 42.9)	42.0) 21.13)
Control group	55.0) 84.10)	56.0) 15.10)	36.0) 85.12)

Table 5 indicates that UP-A+ mindfulness significantly changed depression, CBCL, YAM-I, and YAM-II variables. These meaningful changes were significant and persistent over time.

**Table 5:** Mixed analysis of variance with repeated measures of variables

Variable/Source	SS	Df	MS	F	Sig.	Partial eta
CBCL						
Interaction (time*group)	28.36	1	28.36	37.38	0.01	0.59
Within-subjects (time)	1,216.94	2	608.47	1.03	0.01	0.97
Between subjects (group)	14.79	1	14.79	7.59	0.01	0.23
CDI						
Interaction (time*group)	44.996	1	44.996	312.80	0.01	0.62
Within-subjects (time)	98.416	2	49.20	245.950	0.01	0.90
Between subjects (group)	12.77	1	12.77	25.84	0.01	0.50
YAM-I						
Interaction (time*group)	4.17	1	4.17	10.636	0.01	0.29
Within-subjects (time)	382.43	2	191.21	293.43	0.01	0.92
Between subjects (group)	36.64	1	63.64	52.121	0.01	0.67
YAM-II						
Interaction (time*group)	2.47	1	2.47	7.87	0.01	0.24
Within-subjects (time)	114.09	2	114.09	314.47	0.01	0.92
Between subjects (group)	1.60	1	1.60	5.09	0.01	0.16

SS: Sum of Squares, Df: Degrees of freedom, MS: Mean Square, Sig.: Significance.

Moreover, the results of analysis of variables using Bonferroni pairwise comparisons showed that meaningful changes were observed in the experimental group when pre-test was compared to post-test and to follow-up (p < 0.01).

## Discussion

The aim of this study was to assess the effectiveness of UP-A with an additional mindfulness treatment compared to a UP-A program only for adolescents with depression, anxiety, and behavioral problems. The results of the study show that both treatment methods were able to reduce the adolescents’ emotional problems; but the combination of the unified protocol and mindfulness could lead to greater levels of improvement in anxiety, depression and behavioral problems. In line with these findings, other psychological interventions have shown that unified protocols and mindfulness-based approaches were effective for improving emotional and behavioral problems in adolescents. ^7 , 25^ However, our findings about depression and anxiety, are not consistent with studies that suggest non-significant improvement of depression and anxiety after psychotherapy sessions. ^26 , 27^

To explain how the unified protocol accompanied by mindfulness could improve depression symptoms, it could be pointed out that one of the principles of the unified protocol is cognitive flexibility. ^1^ Evidence shows that reassessing before emotional situations leads to a reduction in the upcoming negative emotions. So reassessment of thoughts before emotional situations occur could facilitate changing those thoughts and balancing the subsequent emotional responses. ^28^ On the other hand, mindfulness meditation forms a state of adaptive observation towards inner experiences and external responses to stimuli, which is characterized by peace and compassion. It does not aim at controlling or avoiding the stimuli or experience, rather it keeps the person in an observational situation. ^13 , 29 , 30^ Therefore, mindfulness helps to regulate emotions and to decrease emotional problems by keeping the person at a psychological distance from the emotions they are experiencing. ^31^

Moreover, the results of this study show that this therapeutic method was able to reduce non-phobic anxiety symptoms in adolescents, which may have stemmed from the principle of identifying emotions and preventing emotional avoidance. ^1^ Investigations demonstrate that people who suffer from emotional disorders try to avoid unexpected or stressful emotional experiences and this avoidance might lead to worsening symptoms. The unified protocol for adolescents encourages acceptance of a range of emotions such as anger or anxiety while discouraging suppression of negative emotions (like non-judgmental awareness); and therefore leads to better emotional adaptation and a reduction in emotional problems. ^32 , 33^ Furthermore, mindful exposure, preventing responding to experiences, and cognitive reconstruction of irritating stimuli as a transient and even positive or meaningful phenomenon may bring about improvements in emotional problems. ^34^

One of the principles of the unified protocol is taking opposite actions, which is a deliberate attempt to act the opposite to how one’s emotion urge; and this principle seems to have been effective for reducing the behavioral problems of the teenagers in the experimental group. According to its effect size, the additional mindfulness content seems to have a low impact on behavioral problems. This principle includes techniques such as behavioral activation, interoceptive exposure, and situational exposure. ^1^ The opposite action technique helps patients to reduce behaviors that are aligned with their emotional preference (avoidance, safety behaviors, and isolation) and motivates them to substitute an opposite action to cope with their emotions. Encouraging the patients to use the opposite action technique might cause unexpected changes in the therapeutic relationship and their enthusiasm, mood, and commitment. Therefore, it is important for the therapist to predict these possibilities and try to speak to adolescents and their caregivers about the importance of being committed to their aim of attending the sessions and utilizing opposite action, despite all the possible changes. ^33^ The low effect size for behavioral problems and specific phobia compared to non-phobic anxiety disorders and depression might therefore be because of a lack of commitment to, or interest in, employing these techniques on the part of adolescents or their parents. Moreover, it could be stated that using behavioral techniques might be beneficial for treating phobia; because coping with phobia requires real exposure, desensitization, and relaxation and these techniques form the foundation of behavioral therapy.

Despite the findings mentioned above, the current research has some limitations which might have restricted generalizability of the findings. Firstly, due to the limited number of participants in our study, the findings should be generalized to other adolescents with caution. Future studies should enroll larger groups of people. Secondly, only self-report scales were used to assess the effectiveness of the treatment and, therefore, it is suggested that future researchers utilize therapist-rated scales. Information generated by techniques like MRI and fMRI might also be beneficial. The third limitation was the two-month follow-up which is considered short for detection of symptom recurrence. Future studies could have longer follow-up periods. Finally, in the current study, the fundamental procedures and active components which could be predictive of therapeutic responses were not clearly determined. For this reason, mediation and moderation analysis might be needed in future investigations.

## Conclusion

On the whole, it can be concluded that additional treatment methods like mindfulness, could enrich the therapy outcomes of the unified protocol for adolescents.
